# Effect of flubendazole on developing stages of *Loa loa in vitro* and *in vivo*: a new approach for screening filaricidal agents

**DOI:** 10.1186/s13071-018-3282-x

**Published:** 2019-01-08

**Authors:** Fanny Fri Fombad, Abdel Jelil Njouendou, Patrick Chounna Ndongmo, Manuel Ritter, Valerine C. Chunda, Haelly M. Metuge, Narcisse Victor T. Gandjui, Peter Enyong, Flobert Njiokou, Achim Hoerauf, Charles D. Mackenzie, Samuel Wanji

**Affiliations:** 1 Parasites and Vectors Biology Research Unit (PAVBRU), Department of Microbiology and Parasitology, Faculty of Science, University of Buea, Buea, Cameroon; 2 Department of Zoology and Animal Physiology, Faculty of Science, University of Buea, Buea, Cameroon; 3 Department of Biomedical Science, Faculty of Health Science, University of Buea, Buea, Cameroon; 4 Institute of Medical Microbiology, Immunology and Parasitology, University Hospital Bonn, Bonn, Germany; 5 Department of Animal Biology and Physiology, Faculty of Science, University of Yaounde I, Yaounde, Cameroon; 6 German Centre for Infection Research (DZIF), Bonn-Cologne partner site, Bonn, Germany; 7 Department of Pathobiology and Diagnostic Investigation, Michigan State University, East Lansing, MI 48824 USA; 8 Filariasis Programmes Support Unit, Liverpool School of Tropical Medicine, Pembroke Place, L3 5QA, Liverpool, UK

**Keywords:** Flubendazole, *Loa loa* L3, Moulting, Motility, *In vitro*, *In vivo*

## Abstract

**Background:**

Loiasis, an often-neglected tropical disease, is a threat to the success of lymphatic filariasis and onchocerciasis elimination programmes in rainforest areas of the central and western Africa. Its control and even its elimination might be possible through the use of a safe macrofilaricide, a prophylactic drug, or perhaps a vaccine. This present study evaluated the effect of flubendazole (FLBZ) on the development of *Loa loa* L3 *in vitro* and *in vivo*.

**Methods:**

Infective stages of *L. loa* were isolated and co-cultured in Dulbecco’s Modified Eagle’s Medium in the presence of monkey kidney epithelial cells (LLC-MK2) feeder cells. FLBZ and its principal metabolites, reduced flubendazole (RFLBZ) and hydrolyzed flubendazole (HFLBZ), were screened *in vitro* at concentrations 0.05, 0.1, 0.5, 1 and 10 μg/ml. The viability of the parasites was assessed microscopically daily for 15 days. For *in vivo* study, a total of 48 CcR3 KO mice were infected subcutaneously with 200 *L. loa* L3 and treated with 10 mg/kg FLBZ once daily for 5 consecutive days. Twenty-four animals were used as control and received L3 and vehicle. They were dissected at 5, 10, 15 and 20 days post-treatment for worm recovery.

**Results:**

The motility of L3 larvae *in vitro* was reduced from the second day of incubation with drugs at *in vivo* plasma concentration levels, with a strong correlation found between reduced motility and increased drug concentration (Spearman’s rho = -0.9, *P* < 0.0001). Except for HFLBZ (0.05 μg/ml and 0.01 μg/ml), all concentrations of FLBZ, HFLBZ and RFLBZ interrupted the moulting of *L. loa* infective larvae to L4. *In vivo*, regardless of the experimental group, there was a decrease in parasite recovery with time. However, at each time point this reduction was more pronounced in the group of animals treated with FLBZ compared to equivalent control. Parasites were recovered from the flubendazole-treated groups only on day 5 post-inoculation at an average rate of 2.1%, a value significantly lower (Mann-Whitney U-test, *U* = 28, *P* = 0.0156) than the average of 31.1% recovered from the control group.

**Conclusions:**

This study reveals the ability of flubendazole to inhibit the development of *L. loa* L3 both *in vitro* and *in vivo,* and in addition validates the importance of *in vitro* and animal models of *L. loa* as tools for the development of drugs against loiasis.

**Electronic supplementary material:**

The online version of this article (10.1186/s13071-018-3282-x) contains supplementary material, which is available to authorized users.

## Background

Loiasis, a neglected tropical disease (NTD), is endemic in the rainforest areas of the central and western Africa [1], where it generally causes minimal pathology [2] that includes localized mild to moderate pruritus, edema and occasional subconjunctival migrations of the adult worm [3]. It is transmitted by blood sucking *Chrysops* flies in which the *Loa loa* microfilariae (mf) develop into infective larvae (L3) and are then inoculated into a human host during the vector’s second blood meal. The infective larvae take 6–12 months to develop into adult worms in the human host; adult worms can survive for more than a decade, during which they migrate through subcutaneous tissues and their females releasing microfilariae into the circulation.

Loiasis has gained prominent attention in the past twenty years because of the cases of severe adverse events (SAEs) occurring in individuals with high microfilaremia treated with ivermectin during mass drug administration (MDA) campaigns in sub-Saharan Africa [4, 5]. High microfilarial load (> 8000 mf/ml) has been reported to be associated with encephalopathy post-ivermectin treatment [4], and is associated with a rapid decrease of circulating mf [6]. This risk of side effects has made MDA with ivermectin unacceptable in areas of co-endemicity where the *L. loa* prevalence exceeds 20% [7], and has therefore compromised onchocerciasis elimination efforts in those areas [8]. Unfortunately, there is currently no recommended drug for the control of loiasis. Diethylcarbamazine (DEC), which was used to treat loiasis, has been reported to induce SAEs in a manner similar to its effect in individuals infected with *Onchocerca volvulus* [9], a filaria often co-endemic in loiasis endemic areas. Reduction in the adverse effects of loiasis would be accelerated if there was a safe macrofilaricidal drug. It has been shown recently that benzimidazole flubendazole can block the transmission of filariae by inhibiting the development of the microfilarial stages of *Brugia* into L3 in an appropriate vector [10]. To date there is no information concerning the effect of this, or other, drugs on the development of L3 into L4. The availability of an *in vitro*, and an *in vivo*, platform where various stages of *L. loa* and other filarial species, such as *Mansonella perstans*, can develop into L4 [11–13] would offer an opportunity to obtain such information. To support development of such a platform, we have here assessed the effect of flubendazole (FLBZ) on the development of *L. loa* L3 *in vitro* and *in vivo*. The results will help to extend the knowledge on the spectrum of filaricidal activities of FLBZ.

## Methods

### Production of *L. loa* L3 larvae

*Loa loa* L3 were obtained from dissected *Chrysops* flies that had previously fed on a consenting microfilariae positive individual at Ediki Forest (South West region, Cameroon) using a previously described protocol [11]. Briefly, engorged *Chrysops* were kept in captivity for 12 days to allow development to the infective stage L3, with the flies then being dissected in Petri dishes containing RPMI 1640 medium (Sigma-Aldrich, St. Louis, USA). The head, thorax and abdomen were teased apart and separated into three different Petri dishes, and all the insect tissue incubated for 20 min to allow migration of any L3 larvae present. A sterile pipette was used to isolate and remove the larvae from each Petri dish, which were then pooled in shallow convex glass dishes followed by transfer into 15 ml centrifuge tubes (Corning, Kennebunk-ME, USA) for purification. Only L3 harvested from the head (where more mature larvae are expected to be found) were used in this present study. The remaining larvae were frozen for future use. The isolated L3 were washed in RPMI 1640 and this suspension concentrated to less than 1 ml by slowly layering it onto the surface of a 15 ml centrifuge tube containing stock iso-osmotic Percoll® (GE Healthcare, Pharmacia, Uppsala, Sweden) and centrifuged (Humax 14k human, Wiesbaden, Germany) at 300× *g* for 10 min.

### *In vitro* culture and assessment of parasite viability

Flubendazole and its principal metabolites, reduced flubendazole (RFLBZ) and hydrolyzed flubendazole (HFLBZ), were obtained from Epichem Pty Ltd (Murdoch, Australia). Stock solution of each drug was prepared at 1 mg/ml in DMSO, and a 10 μl aliquot transferred into a complete culture medium to achieve 10 ml at the concentration of 10 μg/ml. Further dilutions were made in culture medium containing 0.1% DMSO to achieve the final concentrations 1.0, 0.5, 0.1 and 0.05 μg/ml.

Parasites were cultured in Dulbecco’s modified Eagle’s medium (DMEM, Gibco by Life Technologies, Cergy-Pontoise, France) as this medium was previously shown to promote survival and moulting of *L. loa* L3 *in vitro* [13]. This basic medium was supplemented with 100 U/ml penicillin, 100 μg/ml streptomycin, 200 μg/ml neomycin (Gibco by Life Technologies, Cergy-Pontoise, France), 10 μg/ml fluconazole (Sigma-Aldrich, St Louis, USA) and 10% fetal bovine serum (Lonza, Verviers, Belgium). Flat bottom culture plates (48-well) with lids (Corning, USA), pre-coated with LLC-MK2 as previously described [12, 13], were loaded as follows: aliquots of 400 μl of the drug in culture medium at a given concentration, or 0.1% DMSO (negative control), were loaded in each well of the 48-well plate. 10 μl of DMEM containing 15–20 infective larvae were added in each well. Six replicates were created for each drug concentration. The plate was incubated at 37 °C and 5% CO_2_ in a CO_2_ incubator (CO_2_ series Shel Lab, Cornelius, USA), and the L3 motility and moulting scored by two independent trained scientists (blinded to the nature of the treatment) monitored daily for 15 days as previously described [12, 13].

The viability of the parasites was evaluated using their motility as the primary indicator. The motility was scored on a 4-point scale: 0, no movement or immotile; 1, intermittent shaking of head and tail; 2, sluggish (shaking of the whole worm whilst the worms remains in one location in the well); 3, vigorous movement (shaking of the whole worm and with migration from one location in the well to another). The % motility variable was computed based on the scoring system described above, and using the following formula:

$$ \mathrm{Motility}\;\left(\%\right)=\frac{\sum \mathrm{SiNi}}{3.\sum \mathrm{Ni}}\times 100 $$

where Si is the score of point scale i and Ni is the total number of worms at a point scale i [12, 13].

### Experimental animals and *in vivo* studies

Experimental CcR3 BALB/c knockout (KO) mice, 5–6 weeks of age, were shipped from the Institute of Medical Microbiology, Immunology and Parasitology (IMMIP), University Hospital Bonn, Germany, to the Research Foundation for Tropical Diseases and the Environment (REFOTDE), Buea, Cameroon. This strain of animals was selected because eosinophil recruitment was previously shown to be involved in parasite clearing after infection [11]. They were reared and maintained in the laboratory as described previously [11]. Mice were housed in pathogen free cages at the Research Foundation for Tropical Disease and Environment (REFOTDE). Animals were kept at 22 °C and a 12:12 h day:night light cycle. Water and food were provided with a change of autoclaved sawdust bedding twice weekly.

A total of 48 mice (24 in the test group + 24 in the vehicle only group) were used. The L3 obtained from *Chrysops* dissections were concentrated at 200 L3 per 100 μl RPMI 1640 medium. Infection was carried out by subcutaneous injection at the nape of the neck using a 1 ml insulin syringe. Efficiency of inoculation was confirmed by needle washout to check for any remaining parasites.

FLBZ suspension, given subcutaneously, was first homogenised in demineralised water with 0.1% Tween 80 using a Polytron disperser then made up to volume with hydroxyethylcellulose (HEC). The vehicle for the FLBZ suspension was 0.5% w/v HEC in demineralized water containing 0.1% Tween 80. Mice were ear-notched for unique identification and were randomly assigned to test or control groups. Flubendazole was administered subcutaneously by injection of a single dose (10 mg/kg) to all animals in the test group, and 100 μl of its vehicle was administered to each animal in the control group and given 24 h after infection with L3.

Fourteen animals (7 tests and 7 controls) were dissected on each of days 5 and 15 post-drug administration, whilst 10 (5 tests and 5 controls) were dissected on each of days 10 and 20 post-drug administration. Mice were euthanized by exposure to an overdose of CO_2_ [14]. Cardiac blood was first collected by syringe and placed in un-coated 1.5 ml microcentrifuge tubes, and the resulting sera removed and stored at -80 °C for further analysis. A range of organs were gently excised and placed in separate Petri dishes containing RPMI 1640 medium. Muscle tissues were teased gently to ease worm migration into the medium. The sample containing Petri dishes were incubated at 37 °C for 1 h for migration of the *L. loa* worms and then under dissecting microscope (Leica, MDG33/10450123, Singapore) for presence of parasites. The number of worms recovered from the different tissues was recorded on the dissection-recording sheet.

### Data analysis

Raw data were recorded on spreadsheets, and the motility responses generated. Data were further loaded into R version 3.4.1 [15] for statistical analysis and graphical displays. The mean values, standard deviation and other statistical parameters of final records for both *in vitro* and *in vivo* experiments were determined for each experimental group (drug concentration for *in vitro*). The effect of the drugs on the motility of parasites was also assessed by comparing the respective area under the curve (AUC) results using either the first 9 days of culture or 30 days as the time periods for *in vivo* studies. These end time-points were selected to reflect the commencement of moulting, generally characterised by the reduction in motility regardless of the presence of a drug*.* The variable T_90_, defined as the duration at which 90% of the worms were still fully active in the well, was also computed. The values of AUC and T_90_ were expressed as the mean ± standard deviation. The lower the value of AUC or T_90_, the higher the activity of the drug at the concentration or dose indicated. The effects of drug concentrations on parasite viability were compared using non-parametric tests. The Kruskal-Wallis one-way non-parametric ANOVA was used to assess the global significant differences between the median AUC of the various concentrations of each drug. When a difference was detected, Spearman’s rank correlation was used to assess the association between the drug concentration and the AUC of the parasite motility within 9 incubation days. The distribution of median AUC between treated and control groups for the *in vivo* experiment was compared using the Mann-Whitney U-test. Statistical tests were interpreted using a 5% significance level.

## Results

### *In vitro* studies

#### Effect of flubendazole and derivatives on parasites viability

**Fig. 1:**
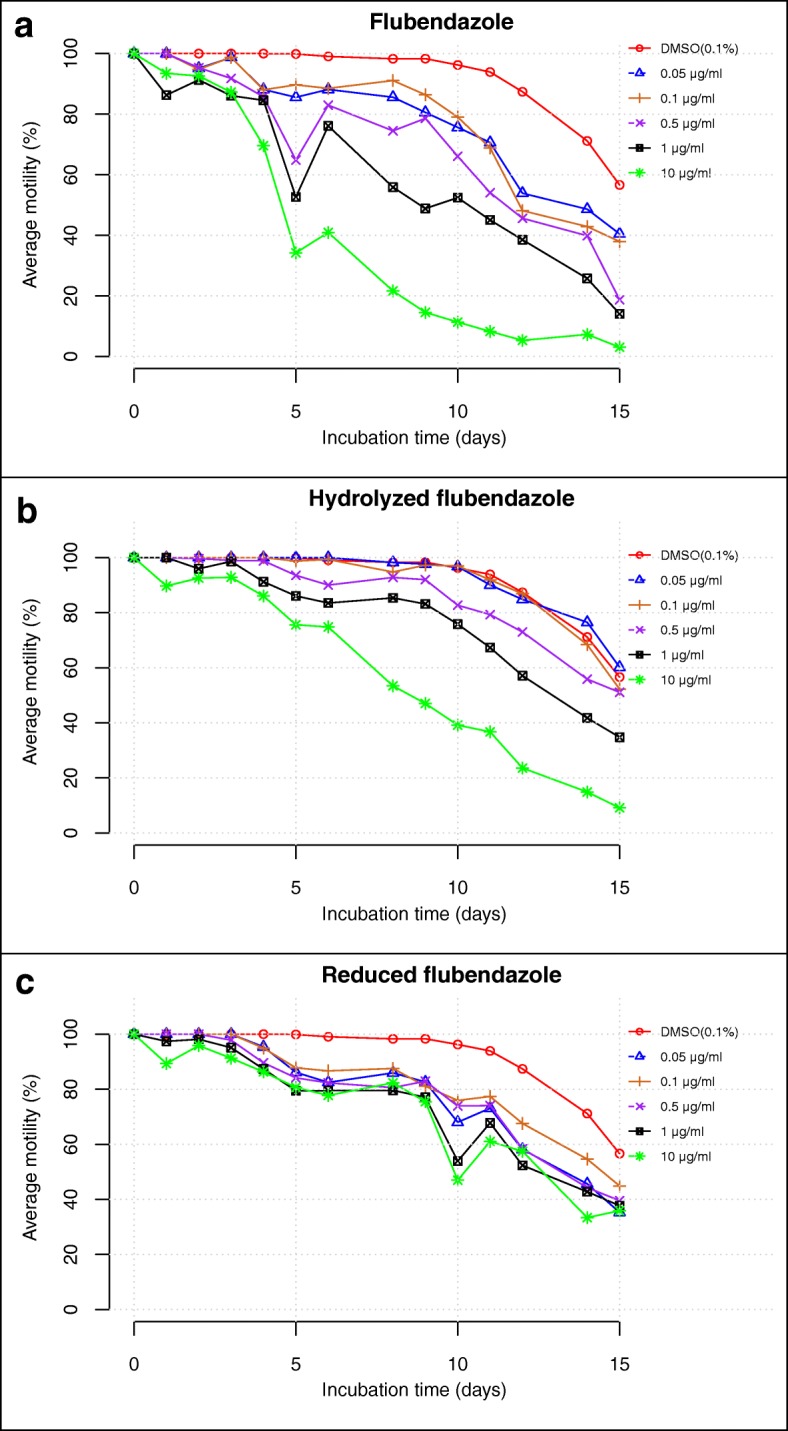
Effects of flubendazole and its derivatives on the motility of *L. loa* L3. **a** Flubendazole (FLBZ). **b** Hydrolysed flubendazole (HFLBZ). **c** Reduced flubendazole (RFLBZ)

*Loa loa* infective larvae remained viable in control wells culture with optimal motility (close to 100%) within the first 9 days of culture, then declined, however remained above 50% (Additional file 1: Table S1, Fig. 1) for 21 days incubation. The motility of L3 larvae *in vitro* was reduced after the second day of incubation with FLBZ at concentrations between 0.05–10 μg/ml; this effect increased with drug concentration. At day 5, the percent motility was 100% in the control (DMSO, 0.1%) compared with less than 40% in treated samples (FLBZ, 10 μg/ml); the latter gradually reduced to a value close to 0% by day 15 (Fig. 1a).

The hydrolyzed derivative of flubendazole (HFLBZ) did not significantly affect parasite motility at concentrations 0.05 or 0.1 μg/ml, compared to the control (DMSO, 0.1%). At the highest drug concentrations (0.5, 1 and 10 μg/ml) however, marked reduction in motility was observed on the third incubation day. Only 10% parasites remain motile by day 15 at 10 μg/ml (Fig. 1b).

Reduced flubendazole also induced reductions in parasite motility regardless of the concentration, and these were generally lower than those seen in control at each time point (Fig. 1c). Although motility reduction was also concentration-dependent, the degree of reduction was lower than those observed with native FLBZ. On day 15, more than 30% of parasites remained actively motile irrespective of the drug concentration; in addition, the motility in the control cultures (0.1% DMSO) also decreased after 9 days of culture.

#### Effects of the concentrations on the activities of flubendazole and its derivatives in vitro

The AUC values for the first 9 days, together with the T_90,_ of each of the three drugs were used to compare the effect of their different concentrations on the parasite motility (Table 1 and Table 2, respectively). At concentrations between 0.05–10 μg/ml, the average AUC ranged between 90.9–58.7, 99.6–78.1 and 91.7–85.7 for FLBZ, HFLBZ and RFLBZ, respectively (Table 1). The Kruskal-Wallis test indicated that irrespective of the drug, the reduction in average AUC varied significantly between concentrations. Interestingly, Spearman’s rank test indicated that the increase in drug concentration is strongly associated with the reduction in AUC of parasite motility.

**Table 1:** Relationship between average AUC and the concentrations of the drugs tested

Drug	Concentration (μg/ml)	Average AUC ± SD	Kruskal-Wallis rank sum test			Spearman’s rank correlation	
			*χ* ^2^	*df*	*P*-value	*rho*	*P*-value
DMSO (0.1%)		99.5 ± 0.4	–	–	–	–	–
FLBZ	0.05	90.9 ± 0.9	19.286	5	0.00170	-0.946	<0.0001
	0.1	92.7 ± 0.6					
	0.5	84.8 ± 2.3					
	1	74.8 ± 0.7					
	10	58.7 ± 0.7					
HFLBZ	0.05	99.6 ± 0.3	17.662	5	0.00340	-0.908	<0.0001
	0.1	98.7 ± 0.4					
	0.5	95.7 ± 0.7					
	1	90.8 ± 1.4					
	10	78.1 ± 0.5					
RFLBZ	0.05	91.7 ± 2.7	18.784	5	0.00211	-0.950	<0.0001
	0.1	92.7 ± 0.8					
	0.5	89.7 ± 0.9					
	1	87.2 ± 0.6					
	10	85.7 ± 0.9					

*Abbreviations*: *AUC* area under the curve, *DMSO* dimethylsulfoxide, *FLBZ* flubendazole, *HFLBZ* hydrolysed flubendazole, *RFLBZ* reduced flubendazole, *SD* standard deviation

**Table 2:** Relationship between average T_90_ and the concentrations of the drugs tested

Drug	Concentration (μg/ml)	Average T_90_ ± SD	Kruskal-Wallis rank sum test			Spearman’s rank correlation	
			*χ* ^2^	*df*	*P*-value	*rho*	*P*-value
DMSO (0.1%)		11.08 ± 0.35	–	–	–	–	–
FLBZ	0.05	5.10 ± 0.1	18.974	5	0.001944	-0.970	<0.0001
	0.1	5.05 ± 0.08					
	0.5	3.15 ± 0.66					
	1	1.97 ± 0.06					
	10	1.10 ± 0.14					
HFLBZ	0.05	10.46 ± 0.18	19.043	5	0.001887	-0.974	<0.0001
	0.1	10.20 ± 0.35					
	0.5	7.21 ± 0.63					
	1	5.03 ± 0.36					
	10	1.90 ± 0.14					
RFLBZ	0.05	5.52 ± 1.01	18.506	5	0.002374	-0.934	<0.0001
	0.1	5.93 ± 0.33					
	0.5	4.87 ± 0.28					
	1	3.54 ± 0.25					
	10	2.85 ± 0.21					

*Abbreviations*: *DMSO* dimethylsulfoxide, *FLBZ* flubendazole, *HFLBZ* hydrolysed flubendazole, *RFLBZ* reduced flubendazole, *SD* standard deviation, *T*_*90*_ Mean duration at which 90% of worm motility were scored 3

The same pattern of result was obtained when considering the average T_90_ values of the various drugs against the experimental concentrations (Table 2), and when concentrations were converted to the logarithmic scale (Additional file 2: Figure S1). At concentrations between 0.05–10 μg/ml, the average T_90_ ranged between 5.10–1.10, 10.46–1.90 and 5.52–2.85 for FLBZ, HFLBZ and RFLBZ, respectively (Table 2). Again, the Kruskal-Wallis test indicated that independent of the drug used, the reduction in average T_90_ significantly varied between concentrations, and high negative correlations were also found between the average T_90_ and the concentrations of each drug.

#### *In vitro* effect of flubendazole and its derivatives on moulting of infective *L. loa* larvae

Except for HFLBZ 0.05 μg/ml and 0.01 μg/ml, all concentrations of FLBZ, HFLBZ and RFLBZ halted the molting of *L. loa* infective larvae to L4. The moulting rate ranged from 23.6 ± 8.8% (DMSO, 0.1%) to 0% for parasites exposed to drugs (Table 3, Additional file 3: Figure S2).

**Table 3:** Percentage moulted parasites in presence of drugs at different concentrations

Drug	Concentration (μg/ml)	Average moulting rate ± SD (%)
DMSO (0.1%)		23.6 ± 8.8
FLBZ	0.05	0
	0.1	0
	0.5	0
	1	0
	10	0
HFLBZ	0.05	14.3 ± 6.0
	0.1	2.4 ± 2.4
	0.5	0
	1	0
	10	0
RFLBZ	0.05	0
	0.1	0
	0.5	0
	1	0
	10	0

*Abbreviation*: *SD* standard deviation

### *In vivo* studies in the CcR3 KO mice model

**Fig. 2:**
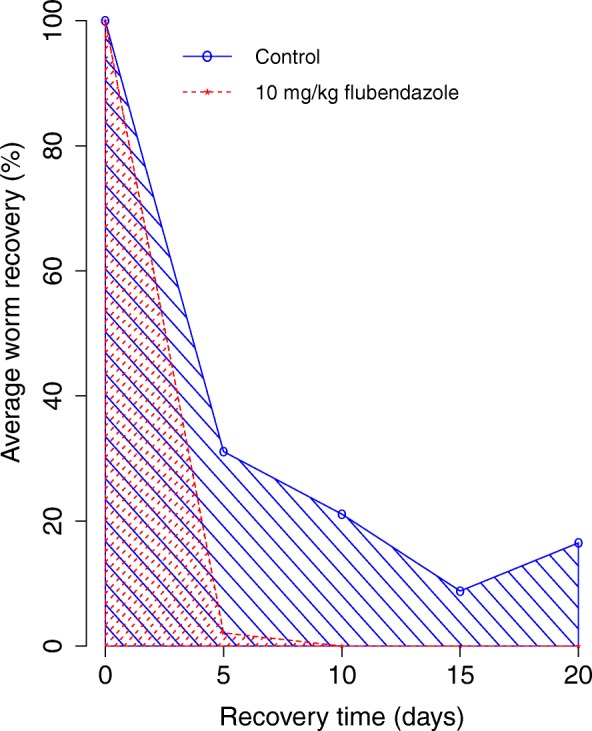
Effect of flubendazole on average *L. loa* recovered from mice within 20 days of infection and treatment

Parasites were recovered from various organs of experimental animals and counted (Additional file 4: Table S2). The major sites of worm recovery were the subcutaneous and muscle tissue. The average percentage worm recovery and other statistical parameters of the two experimental groups of animals within 30 days are provided in Table 4. Regardless of the treatment group, there were decreases in parasite recovery with time. However, this decrease was more pronounced in the group of animals treated with FLBZ than with the control at each time point. In the FLBZ-treated group, parasites were recovered only on day 5 post-inoculation at an average rate of 2.1%. This value was significantly lower (Mann-Whitney U-test, *U* = 28, *P* = 0.0156) than the average of 31.1% recovered in the control group. The difference between the AUC for both groups presented in Table 4 and illustrated in Fig. 2 were also statistically significant (Mann-Whitney U-test, *U* = 4, *P* = 0.01052).

**Table 4:** Summary statistics of worm recovery in CcR3 KO mice within 30 days

Experimental groups	Statistics	Recovery time (days)				AUC (%)
		5	10	15	20	
Control (*n* = 7)^a^	Mean	31.1	21.5	8.8	16.5	23.9
	Geometric mean	22.4	11.8	4.2	7.1	21.3
	Median	35.5	14.5	5.5	12.0	20.8
	SD	18.05	26.1	9.6	19.6	11.9
10 mg/kg FLBZ (s.c., single dose, *n* = 7)^a^	Mean	2.1	0	0	0	8.7
	Median	2.5	0	0	0	8.8
	Geometric mean	1.5	0	0	0	8.7
	SD	2.1	0	0	0	0.4
Mann-Whitney rank sum test	*U*	28	–	–	–	4
	*P*-value	0.0156	–	–	–	0.01052

*Abbreviations*: *AUC* area under the curve, *n* number of animal per group, *FLBZ* flubendazole, – not computed, *SD* standard deviation

^a^Seven animals were dissected at each time point except on days 10 and 20, where 5 were dissected

## Discussion

Loiasis remains an important public health issue, and treatment options are limited due to severe adverse events [4, 5]. However, it has been shown that FLBZ, know essentially as a macrofilaricidal agent drug [16, 17], exhibits little or no microfilaridal activity against several filarial species, such as *Brugia pahangi* [18], *Onchocerca lienalis* [19] and *L. loa* [20], and its capacity in eliminating adult worms has been elucidated in many studies for filarial species such as *B. pahangi* [18, 21], *Breinlia booliati* [22] and *Onchocerca ochengi* [23]. In this study, we showed that FLBZ reduces worm motility from 80.5% to 14.5% by 9 days after incubation at concentrations of 0.05–10 μg/ml, using the recently published filarial *in vitro* culture system that has been shown to promote development and moulting of infective stages of *L. loa* [13] and *M. perstans* [12]. Reduction in motility was found to be concentration-dependent. Similar activities were observed with the reduced metabolite of FLBZ with motility reduction of 82.6% to 75.4% by day 9 at concentrations of 0.05–10 μg/ml. Previous investigation of the pharmacokinetics of FLBZ and its main metabolites has revealed that their pharmacokinetics parameters are species-dependent. In lambs and adult sheep, FLBZ is present at very low concentrations (Cmax = 0.04 μg/ml), close to the lowest concentration tested in this study, while RFLBZ is identified as the main metabolite (Cmax = 0.14 μg/ml), and HFLBZ is present in trace amounts [24, 25], with a Cmax far below the lowest concentration tested here. The reduced metabolite of flubendazole was also reported to be the major metabolite in mice [26]. In pigs, however, the hydrolysed metabolite of flubendazole represent 97% of the total plasmatic drug after oral administration [27]. In rats and jirds, a completely different profile has been reported, with the parental drug being the major drug detected, followed by its hydrolysed derivative against trace amount of RFLBZ [28].

Previous studies with a range of helminths indicated that RFLBZ is the main, and perhaps the sole, active metabolite, with HFLBZ having no anthelminthic activity [29–31]. However, our findings here, in addition to other recent observations [20], indicate that FLBZ and its derivatives display heterogenic patterns of activity depending on the species of helminth and the stage being considered. At concentrations of 1 μg/ml and above, there was a clear reduction in *L. loa* larvae motility with FLBZ, and with its reduced metabolite. HFLBZ was also active, and at comparable concentrations, this metabolite seen to be more active *in vitro* (CR_50_ = 8.1 μg/ml) against microfilaria of the same species than RFLBZ (CR_50_ = 17.5 μg/ml) and FLBZ (CR_50_ = 21 μg/ml) [20]. In addition, at these concentrations worms did not appear to moult in the presence of FLBZ and RFLBZ, but did in wells treated with HFLBZ; the percentage of moulted worms in these decreased as a result of increase in drug concentration. These latter observations are not easily interpreted but may be due the parallel effects of stress and survival attempts by these worms under these conditions; the true reason would require further investigation. These present observations contrast with previous findings on microfilariae of *L. loa*, where it was observed that the inhibitory effect of parasite viability was more pronounced in the presence of HFLB rather than FLBZ and its reduced metabolite [20]. The motility of the control cultures was found to be reduced after the 9th day of culture; this period coincided with the starting point of moulting and corroborated our previous observations [13].

Our result here concerning the inhibition of motility *in vitro* indicate that FLBZ and its major metabolites are not acutely toxic to the developing stages of *L. loa*. However, arguably the most important effect observed in the present study is the inhibition of moulting, and the consequent prevention of the development of infective stages to the adult form. Previous studies on the enzymology of the cuticle in parasitic nematodes have highlighted the inhibition of enzymes involved in parasite escheatment as promising approach for drug/vaccine development [32]. Thus, in addition in the inhibition of motility, the inhibition of moulting is a likely useful indicator for determining the potential of filaricides as agents for blocking disease transmission.

The *in vivo* findings in this present study added important information regarding the efficacy of flubendazole and its metabolites. Following administration of a single dose of FLBZ (10 mg/kg) to infected mice, parasites were recovered only on day 5 post-inoculation at an average rate of 2.1%. The recovery rate in the flubendazole-treated group was significantly lower than the average of 31.1% recovered in the control group, indicating that this drug inhibits parasite development. A difference in parasite survival between the two groups of animals was also confirmed by the difference in their AUCs. As observed *in vitro*, the residual FLBZ and its major metabolite were shown to be more active in inhibiting both parasite growth and survival; this underscores the possibility that this agent may play an important role in blocking parasite development *in vivo*.

## Conclusions

This study has revealed that FLBZ can inhibit the development of *L. loa* L3 both *in vitro* and *in vivo*. To our knowledge, this is the first time that FLBZ has been demonstrated to inhibit the moulting of infective larvae of *L. loa* into L4. These findings highlight the potential of FLBZ in blocking transmission of *L. loa.* The study also demonstrates the added value of using both *in vitro* and *in vivo* platforms when assessing the efficacy of drugs on developing stages of *L. loa*, and thus provide an additional approach for the screening of filaricides.

## Additional files

Additional file 1: **Table S1.** Average and standard deviation value of the % motility of L3 in presence of tested drugs at different scoring days. (XLSX 17 kb)

Additional file 2: **Figure S1.** Relationship between average AUC, average T_90_ and the logarithm of concentrations of FLBZ and derivatives. (PDF 5 kb)

Additional file 3: **Figure S2.** Potential of HFLBZ in inhibiting the moulting of *L. loa* L3 to L4. No worm moulted at concentrations of HFLBZ greater than 0.5 μg/ml and in presence of FLBZ or RFLBZ at concentrations between 0.05–10 μg/ml and therefore these drugs are not included in the figure. Spearman’s rank correlation: *rho* = -0.9006, *P* < 0.0001. (PDF 4 kb)

Additional file 4: **Table S2.** Percentage worm recovery per organ of the mice. (DOCX 14 kb)

Additional file 5: **Table S3.** Dataset for *in vitro* effect of flubendazole and derivatives at different concentrations on *Loa loa* L3. (CSV 48 kb)

Additional file 6: **Table S4.** Dataset for *in vivo* effect of flubendazole 10 mg/kg FBZ (s.c., single dose) on the development of *Loa loa* infective larvae in CcR3KO/KO mice. (CSV 4 kb)

## Abbreviations

AUC: Area under the curve
CCM: Complete culture medium
DMEM: Dulbecco’s modified Eagle’s medium
DMSO: Dimethyl sulfoxide
DNDi: Drug for Neglected Diseases initiative
FLBZ: Flubendazole
HFLBZ: Hydrolyzed flubendazole
LLC-MK2: Monkey kidney epithelial cells
Mf: Microfilaria
RFLBZ: Reduced flubendazole
RPMI: Roswell Park Memorial Institute
T_90_: Mean duration at which 90% of worm motility were scored 3

## References

[1] Padgett JJ, Jacobsen KH (2008). Loiasis: African eye worm. Trans R Soc Trop Med Hyg.

[2] Pinder M (1988). *Loa loa* - a neglected filaria. Parasitol Today.

[3] Mongin A (1770). Observations sur un ver trouvé sous la conjonctive à Maribarou, île Saint-Dominique. J Med Chir Pharm Paris.

[4] Gardon J, Gardon-Wendel N, Demanga N, Kamgno J, Chippaux J-P, Boussinesq M (1997). Serious reactions after mass treatment of onchocerciasis with ivermectin in an area endemic for *Loa loa* infection. Lancet.

[5] Chippaux J-P, Boussinesq M, Gardon J, Gardon-Wendel N, Ernould J-C (1996). Severe adverse reaction risks during mass treatment with ivermectin in loiasis-endemic areas. Parasitol Today.

[6] Kazura J, Greenberg J, Perry R, Weil G, Day K, Alpers M (1993). Comparison of single-dose diethylcarbamazine and ivermectin for treatment of bancroftian filariasis in Papua New Guinea. Am J Trop Med Hyg.

[7] Boussinesq M, Gardon J, Kamgno J, Pion S, Gardon-Wendel N, Chippaux J-P (2001). Relationships between the prevalence and intensity of *Loa loa* infection in the Central Province of Cameroon. Ann Trop Med Parasitol.

[8] Boussinesq M (2006). Loiasis. Ann Trop Med Parasitol.

[9] Francis H, Awadzi K, Ottesen EA (1985). The mazzotti reaction following treatment of onchocerciasis with diethylcarbamazine: clinical severity as a function of infection intensity. Am J Trop Med Hyg.

[10] O’Neill M, Njouendou JA, Dzimianski M, Burkman E, Ndongmo PC, Kengne-Ouafo JA (2018). Potential role for flubendazole in limiting filariasis transmission: observations of microfilarial sensitivity. Am J Trop Med Hyg.

[11] Tendongfor N, Wanji S, Ngwa JC, Esum ME, Specht S, Enyong P (2012). The human parasite *Loa loa* in cytokine and cytokine receptor gene knock out BALB/c mice: survival, development and localization. Parasit Vectors.

[12] Njouendou AJ, Ritter M, Ndongmo WPC, Kien CA, Narcisse GTV, Fombad FF (2017). Successful long-term maintenance of *Mansonella perstans* in an *in vitro* culture system. Parasit Vectors.

[13] Zofou D, Fombad FF, Gandjui NVT, Njouendou AJ, Kengne-Ouafo AJ, Chounna Ndongmo PW (2018). Evaluation of *in vitro* culture systems for the maintenance of microfilariae and infective larvae of *Loa loa*. Parasit Vectors.

[14] Ajendra J, Specht S, Neumann A-L, Gondorf F, Schmidt D, Gentil K (2014). ST2 deficiency does not impair type 2 immune responses during chronic filarial infection but leads to an increased microfilaremia due to an impaired splenic microfilarial clearance. PLoS One..

[15] Development Core Team R (2017). R: A Language and Environment for Statistical Computing. 3.4.1 edn. Vienna.

[16] Mackenzie CD, Geary TG (2011). Flubendazole: a candidate for a field usable macrofilariacide for lymphatic filariasis and onchocerciasis field programs. Expert Rev Anti Infect Ther.

[17] Geary TG, Mackenzie CD (2011). Progress and challenges in the discovery of macrofilaricidal drugs. Expert Rev Anti Infect Ther.

[18] Denham DA, Samad R, Cho SY, Suswillo RR, Skippins SC (1979). The anthelmintic effects of flubendazole on *Brugia pahangi*. Trans R Soc Trop Med Hyg.

[19] Townson S, Dobinson A, Connelly C, Muller R (1988). Chemotherapy of *Onchocerca lienalis* microfilariae in mice: a model for the evaluation of novel compounds for the treatment of onchocerciasis. J Helminthol.

[20] Njouendou AJ, Fombad FF, O’Neill M, Zofou D, Nutting C, Ndongmo PC (2018). Heterogeneity in the *in vitro* susceptibility of *Loa loa* microfilariae to drugs commonly used in parasitological infections. Parasit Vectors.

[21] Van Kerckhoven I, Kumar V (1988). Macrofilaricidal activity of oral flubendazole on *Brugia pahangi*. Trans R Soc Trop Med Hyg.

[22] Mak JW (1981). Antifilarial activity of mebendazole and flubendazole on *Breinlia booliati*. Trans R Soc Trop Med Hyg.

[23] Bronsvoort B, Makepeace B, Renz A, Tanya V, Fleckenstein L, Ekale D (2008). UMF-078: a modified flubendazole with potent macrofilaricidal activity against *Onchocerca ochengi* in African cattle. Parasit Vectors.

[24] Krizova V, Nobilis M, Pruskova L, Chladek J, Szotakova B, Cvilink V (2009). Pharmacokinetics of flubendazole and its metabolites in lambs and adult sheep (*Ovis aries*). J Vet Pharmacol Ther.

[25] Moreno L, Alvarez L, Mottier L, Virkel G, Bruni SS, Lanusse C (2004). Integrated pharmacological assessment of flubendazole potential for use in sheep: disposition kinetics, liver metabolism and parasite diffusion ability. J Vet Pharmacol Ther.

[26] Ceballos L, Elissondo M, Bruni SS, Denegri G, Alvarez L, Lanusse C (2009). Flubendazole in cystic echinococcosis therapy: pharmaco-parasitological evaluation in mice. Parasitol Int.

[27] Ceballos L, Alvarez L, Mackenzie C, Geary T, Lanusse C (2015). Pharmacokinetic comparison of different flubendazole formulations in pigs: a further contribution to its development as a macrofilaricide molecule. Int J Parasitol Drugs Drug Resist.

[28] Ceballos L, Mackenzie C, Geary T, Alvarez L, Lanusse C (2014). Exploring the potential of flubendazole in filariasis control: evaluation of the systemic exposure for different pharmaceutical preparations. PLoS Negl Trop Dis.

[29] Ceballos L, Elissondo C, Bruni SS, Denegri G, Lanusse C, Alvarez L (2011). Comparative performance of flubendazole and albendazole in cystic echinococcosis: Ex vivo activity, plasma/cysts disposition and efficacy in infected mice. Antimicrob Agents Chemother.

[30] Alvarez L, Moreno G, Moreno L, Ceballos L, Shaw L, Fairweather I, Lanusse C (2009). Comparative assessment of albendazole and triclabendazole ovicidal activity on *Fasciola hepatica* eggs. Vet Parasitol.

[31] Urbizu L, Confalonieri A, Bruni SS, Lanusse C, Alvarez LI (2012). Nematodicidal activity of flubendazole and its reduced metabolite on a murine model of *Trichinella spiralis* infection. Chemotherapy.

[32] Page AP, Stepek G, Winter AD, Pertab D (2014). Enzymology of the nematode cuticle: A potential drug target?. Int J Parasitol Drugs Drug Resist.

[33] Hollands C (1986). The Animals (Scientific Procedures) Act 1986. Lancet.

