# Supplementary figures and images for: Effect of flubendazole on developing stages of Loa loa in vitro and in vivo: a new approach for screening filaricidal agents
Source: Parasit Vectors. 2019 Jan 8;12:14. doi: 10.1186/s13071-018-3282-x (PMC6323797; doi:10.1186/s13071-018-3282-x)

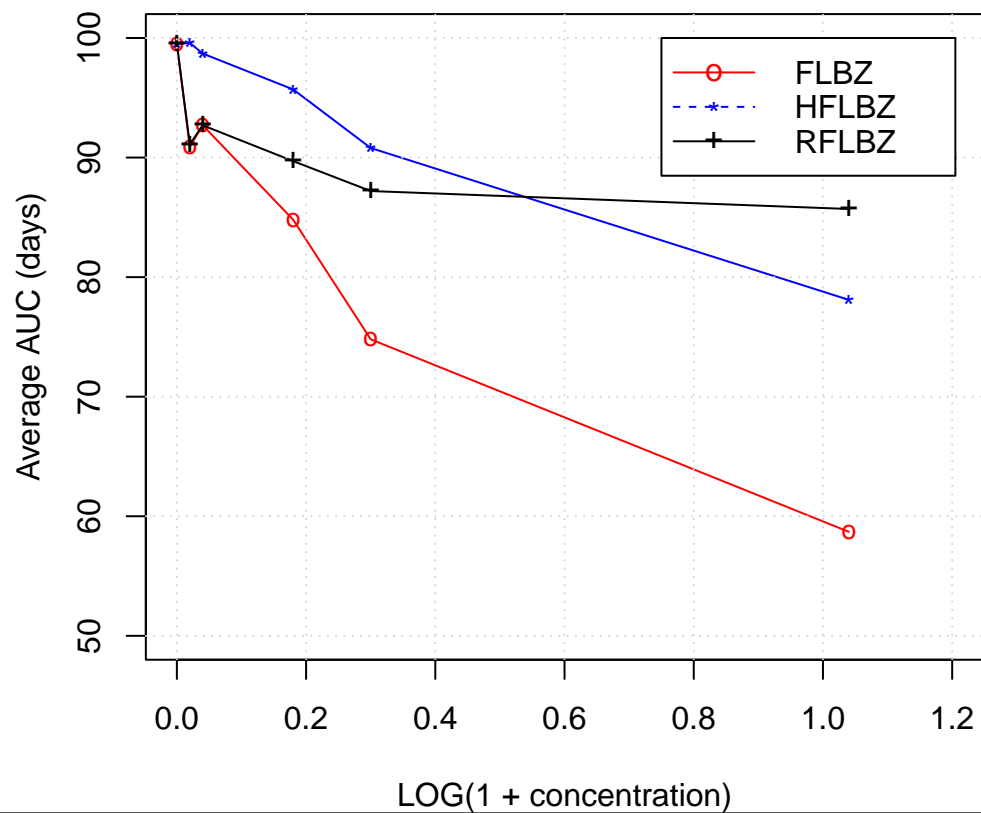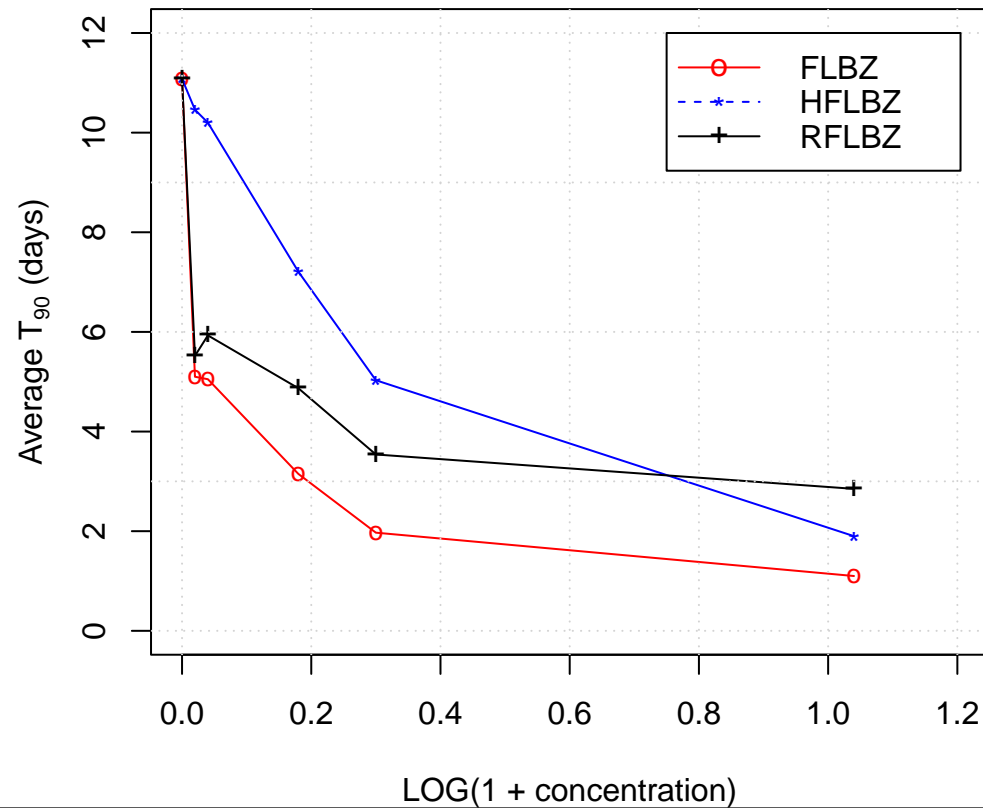

Supplement: Supplementary file 2 — Figure S1. Relationship between average AUC, average T90 and the logarithm of concentrations of FLBZ and derivatives. (PDF 5 kb) [file 13071_2018_3282_MOESM2_ESM.pdf]

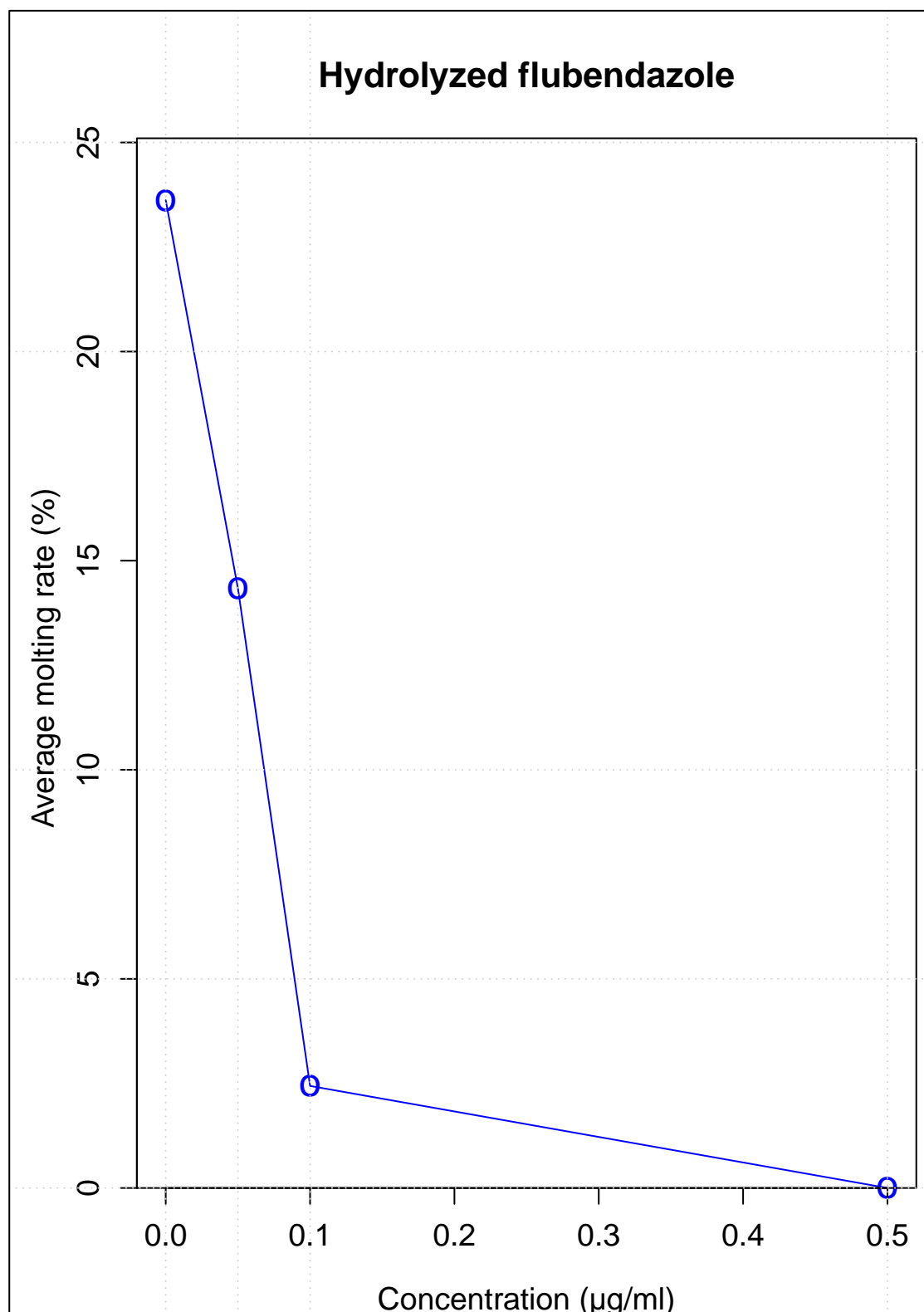

Supplement: Supplementary file 3 — Figure S2. Potential of HFLBZ in inhibiting the moulting of L. loa L3 to L4. No worm moulted at concentrations of HFLBZ greater than 0.5 μg/ml and in presence of FLBZ or RFLBZ at concentrations between 0.05–10 μg/ml and therefore these drugs are not included in the figure. Spearman’s rank correlation: rho = -0.9006, P < 0.0001. (PDF 4 kb) [file 13071_2018_3282_MOESM3_ESM.pdf]
