# Supplementary material for: Effect of flubendazole on developing stages of Loa loa in vitro and in vivo: a new approach for screening filaricidal agents
Source: Parasit Vectors. 2019 Jan 8;12:14. doi: 10.1186/s13071-018-3282-x (PMC6323797; doi:10.1186/s13071-018-3282-x)
Supplement: Supplementary file 4 — Table S2. Percentage worm recovery per organ of the mice. (DOCX 14 kb) [file 13071_2018_3282_MOESM4_ESM.docx]

**Additional file** **4: Table S2.** Percentage worm recovery per organ of the mice

| Dose | Time (days) | Sub-cutaneous | Muscles | Intestines | Pleural cavity | Others |
| --- | --- | --- | --- | --- | --- | --- |
| 10 mg/kg FLBZ (s.c., single dose, *n* = 7*) | 5 | 1.2 | 1 | 0 | 0 | 0 |
|  | 10 | 0 | 0 | 0 | 0 | 0 |
|  | 15 | 0 | 0 | 0 | 0 | 0 |
|  | 20 | 0 | 0 | 0 | 0 | 0 |
|  |  |  |  |  |  |  |
| Control (*n* = 7*) | 5 | 13.8 | 16.2 | 0.2 | 0 | 1.1 |
|  | 10 | 6.3 | 15.1 | 0.1 | 0 | 0 |
|  | 15 | 2.2 | 6 | 0 | 0.3 | 0.3 |
|  | 20 | 8 | 7.8 | 0 | 0.2 | 0.6 |
|  |  |  |  |  |  |  |

* seven (7) animals were dissected at each time point except on days 10 and 20 where 5 were dissected.
